# A rare case of polyorchidism in a 40-year-old man. A case report

**DOI:** 10.1016/j.amsu.2021.102389

**Published:** 2021-05-12

**Authors:** Muhammad Mazketly, Owais Aleter, Muhamad Zakaria Brimo Alsaman, Bashar Bazkke, Majed Eddin Jouda, Alae Kayyali

**Affiliations:** a Faculty of Medicine, University of Aleppo, Aleppo, Syria; b Radiology Department, Aleppo University Hospital, Aleppo, Syria

**Keywords:** Polyorchidism, Triorchidism, Supernumerary testis, Urology, Radiology, Urological imaging

## Abstract

**Introduction:**

and importance Polyorchidism is defined as the presence of three testes or more. Approximately, there are about 200 cases of polyorchidism in the medical literature. In the past, surgical treatment was done but now with imaging studies, less aggressive approach is recommended.

Here we present a case of 40-year-old man who was diagnosed incidentally with polyorchidism in the right hemiscrotum which is quite unusual in this age.

**Case presentation:**

A 40-year-old man presented to the emergency department with a swelling and pain in the left hemiscrotum. In palpation, we noticed a scrotal mass in the right hemiscrotum. His parents had first noticed a scrotal mass when he was two years old and was incorrectly diagnosed as hydrocele by an unauthorized practitioner.

In the left hemiscrotum, Doppler confirmed acute epididymitis diagnosis that was treated conservatively with antibiotics and NSAIDs. In the right hemiscrotum, MRI showed that the lump had separate epididymis and shared a common vas deferens with the right testis, which confirmed the diagnosis of supernumerary testis and the patient underwent a follow-up ultrasound after a month and after six months of his presentation.

**Discussion:**

Triorchidism is the most common type of polyorchidism. Polyorchidism is diagnosed incidentally hence it is asymptomatic. There are many types of Polyorchidism and tow classification have been described. When the patient is asymptomatic the concentrative treatment is recommended.

**Conclusion:**

Polyorchidism is a rare congenital anomaly in the genitourinary tract. It is diagnosed incidentally. Ultrasound or MRI are used to diagnose polyorchidism cases.

## Highlights

• This Case Report is about a rare condition (polyorchidism) in a 40-year-old man which is very unusual in this age.
• Diagnosis has been made by imaging studies only. No need for aggressive treatment in uncomplicated cases.
• This case report provided a brief review about the classification of Polyorchidism and other differential diagnosis.

## 1 Introduction

Polyorchidism or supernumerary testis (SNT) is a rare congenital anomaly in the genitourinary Tract. It is defined as the presence of three testes or more.

Polyorchidism has many forms; Triorchidism (three testes) is the most common type. Bilateral double testis (4 testes) is a rare type [1].

Ahlfeld first described polyorchidism histologically in 1880. It was first reported by Lane in 1895 as clinical case [2].

Approximately, there are about 200 cases of polyorchidism in the medical literature [3].

Most cases of Polyorchidism are asymptomatic and are diagnosed incidentally. About 65% of polyorchidism cases were reported on left side with median age 17 years [1].

Ultrasound or MRI help in diagnosis and follow polyorchidism patients [1].

Here we present a case of 40-year-old man who was diagnosed incidentally with polyorchidism in the right hemiscrotum which is quite unusual in this age. This case report has been reported in line with the SCARE Criteria [4].

## 2 Case Presentation

A 40-year-old man presented to the emergency department with a swelling and pain in the left hemiscrotum with no history of trauma or surgical procedures. Therefore, he was referred to the urology department. Drugs and Family history are unremarkable, the patient does not take any medication and he does not recall any type of allergy. He smokes one pack-year and does not drink alcohol and works as trader.

Physical examination indicated an acute left epididymitis. In palpation, we noticed a scrotal mass in the right hemiscrotum.

His parents had first noticed scrotal mass when he was two years old and was diagnosed incorrectly as hydrocele by an unauthorized practitioner. He had not undergone any further investigation.

**Fig. 1:**
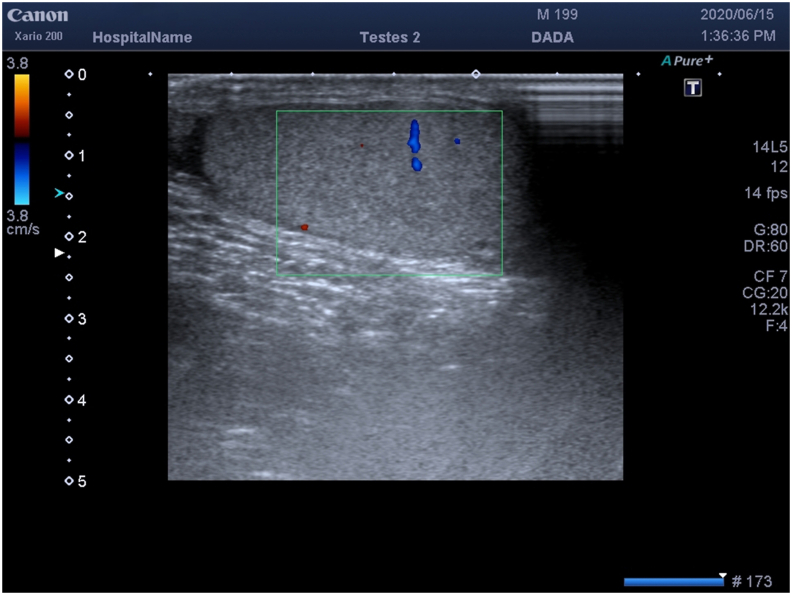
Color Doppler image shows blood flow and echotexture of the supernumerary testis, which is similar to the other testes. (For interpretation of the references to colour in this figure legend, the reader is referred to the Web version of this article.)

We performed US, which showed normal left testis and enlarged left epididymis with hypoechoic. In addition, we performed Doppler evaluation to demonstrate blood flow, which was increased. Therefore, we confirmed acute epididymitis diagnosis that was treated conservatively with antibiotics and NSAIDs. In the right hemiscrotum, Color Doppler ultrasound evaluation revealed oval lump with echotexture and blood flow similar to the ipsilateral testis (Fig. 1).

We put Triorchidism and adenomatoid tumor as differential diagnosis for this lump.

The Serum tumor markers were in the normal range. (AFP; 1.44 IU ML−1), and (beta-HCG; 0.5 MUI ML−1).

**Fig. 2:**
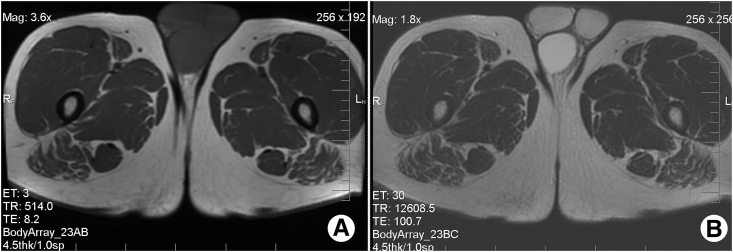
(A) Transverse T1 weighted imagine of the scrotum show the supernumerary testis posterior to the right testis and bigger than other testes, but with signal intensity similar to them. (B) Transverse T2 weighted imagine show that the supernumerary testis has higher signal intensity than other testes without any focal changes.

MRI showed that the lump had separate epididymis and shared a common vas deferens with the right testis, that was not obvious on Doppler ultrasound evaluation, which confirmed the diagnosis of supernumerary testis (Triorchidism). The supernumerary testis had intermediate signal intensity on T1-weighted images and high signal intensity on T2-weighted images and that is the same of normal testes (Fig. 2).

The left testis measured 43 mm in Long axis, the right testis measured 46 mm, and the supernumerary testis measured 58 mm and situated posteriorly and inferiorly to the right testis.

No Histological examination was performed because the patient refused the biopsy.

The classification of the Supernumerary testis was type A2 according to Bergholz and wenke (2009) classification and type C according to Leung classification.

The patient underwent a follow-up ultrasound after a month and after six months of his presentation. Then we put the patient on regular follow-up yearly. The patient is still radiologically stable 6 month after his presentation, with his radiologic follow-up the patient has good prognosis, and he did not have any complications as the clinician expected.

## 3 Discussion

Testis begins to form around 6th week of gestiaon from genital ridge which is a small bulge on the dorsal coelomic wall that contain migratory primordial germ cells. There are many theories that point to the cause of polyorchidism such as anomalous appropriation of cells, duplication or transverse vs longitudinal division of the urogenital ridge, incomplete degeneration of a portion of the mesonephros and development of peritoneal bands [1,5].

Triorchidism is the most common type of polyorchidism. However, bilateral double polyorchidism (4 testicles) had been reported [6]. The left side is the most common place for supernumerary testicle, inguinal canal is also a possible location [1]. In comparison, the supernumerary testicle in our patient is in the right hemiscrotum.

The median age of polyorchidism cases is 17 [1] and approximately, there are about 200 cases of polyorchidism in the medical literature [3].

Here we present a case of 40-year-old man who was diagnosed incidentally with polyorchidism in the right hemiscrotum which is quite unusual in this age.

Polyorchidism is diagnosed incidentally hence it is asymptomatic. However, it could be symptomatic when it is associated with cancer [1]. In our case, the patient was complaining of acute epididymitis in the left scrotum.

Based on Leung classification there are four types of polyorchidism: Type A. the supernumerary testicle has neither epididymis nor vas deferens and has no connection to the other testicles. Type B. the supernumerary testicle shares the same epididymis and vas deferens of the other testicles. Type C. the supernumerary testicle has separate epididymis but shares a vas deferens with the other testicles. Type D. the supernumerary testicle has separate epididymis and vas deferens [7].

Furthermore, in a meta-analysis study another classification has been described: Type A when it is connected to vas deferens with subgroups: A1 separate epididymis and vas deferens. A2 separate epididymis but shares vas deferens with other testicle A3 shares epididymis and vas deferens with other testicle. A4 separate vas deferens but shares epididymis with other testicle. It is classified as Type B when it has no connection to vas deferens with subgroups: B1 separate epididymis. B2 only testicular tissue. The most common type is A3 [1].

In our case, the classification of the Supernumerary testis was type A2 according to Bergholz and Wenke (2009) classification and type C according to Leung classification.

In the same meta-analysis, 73% of the cases were diagnosed histologically which is quite different in our case where we only diagnosed the case with imaging studies [1]. This also carry a less aggressive approach to the patient.

Differential diagnoses for polyorchidism are: encysted hydrocele, spermatocele or varicocele, Morgagnian cyst or testicular neoplasm, therefore, physical examination alone is not enough to diagnose polyorchidism. Imaging study like Ultra Sound, Doppler and MRI are essential for diagnosis, US is the most common imaging study and can confirm the diagnosis, MRI is less common [1,8].

We did not confirm the diagnosis by histological examination because the patient refused the biopsy and there is no need for it because there was no neoplastic signs and the imagining study alone confirmed polyorchidism [1].

The treatment of polyorchidism is still debated. In the past, Surgical removal of supernumerary testicle was preformed but the presence of imaging study changed the way we treat polyorchidism, when the patient is asymptomatic Type A (our patient) the concentrative treatment is recommended, but the patient should be monitored regularly because there is an increased number of malignancy in case of polyorchidism. If the malignancy was confirmed a surgical removal is recommended [1]. Based on that our patient is under concentrative treatment with close regular monitoring.

## 4 Conclusion

Polyorchidism is a rare congenital anomaly in the genitourinary tract. However, it should be considered as a differential diagnosis of scrotal mass despite its rarity. It is diagnosed incidentally. Ultrasound or MRI are being using to diagnose polyorchidism cases.

## Patient consent

Written informed consent was obtained from the patient for publication of this case report and accompanying images. A copy of the written consent is available for review by the Editor-in-Chief of this journal on request.

## Ethics approval and consent to participate

Not applicable.

## Availability of data and materials

Data used on this case report are available from corresponding author on reasonable request.

## Authors' contributions

AK and MEJ: Revision of the manuscript. MM, MZBA and BB: Drafting of the manuscript. OA: Conception and design of the study. AK: Approval of the final version of the manuscript.

All authors confirm that they have read and approved the final manuscript.

## Guarantor

Owais Aleter.

## Provenance and peer review

Not commissioned, externally peer-reviewed.

## Consent for publication

The patient signed consent form to publish this case with the figures.

## Ethical approval

The patient was informed and approved the manuscript.

## Please state any sources of funding for your research

None.

## Consent

Approved

## Please state any conflicts of interest

None.

## Registration of research studies

1. Name of the registry:

2. Unique Identifying number or registration ID:

3. Hyperlink to your specific registration (must be publicly accessible and will be checked):

## Guarantor

Owais Aleter.

## Funding

No funding was obtained for this case report.

## Declaration of competing interest

Authors declare they have no conflict of interest.

## List of abbreviations

SNT: Supernumerary testis
MRI: Magnetic resonance imaging
US: Ultrasound
AFP: alpha-fetoprotein
Beta-HCG: Beta human chorionic gonadotropin
