# Supplementary material for: A rare case of polyorchidism in a 40-year-old man. A case report
Source: Ann Med Surg (Lond). 2021 May 12;66:102389. doi: 10.1016/j.amsu.2021.102389 (PMC8144338; doi:10.1016/j.amsu.2021.102389)
Supplement: Multimedia component 1 [file mmc1.docx]

| **SCARE 2020 Checklist** | | | |
| --- | --- | --- | --- |
| **Topic** | **Item** | **Checklist Item Description** | **Page Number** |
| **Title** | **1** | - A rare case of polyorchidism in a 40-year-old man. A case report | 1 |
| **Key Words** | **2** | - Polyorchidism, Triorchidism, Supernumerary testis, Urology, Radiology, Urological Imaging | 1 |
| **Abstract** | **3a** | Introduction and Importance   - Polyorchidism is defined as the presence of three testes or more. Approximately, there are about 200 cases of polyorchidism in the medical literature. In the past, surgical treatment was done but now with imaging studies, less aggressive approach is recommended. - Here we present a case of 40-year-old man who was diagnosed incidentally with polyorchidism in the right hemiscrotum which is quite unusual in this age. | 1 |
|  | **3b** | Case Presentation   - A 40-year-old man presented to the urology department with a swelling and pain in the left hemiscrotum. In palpation, we noticed a scrotal mass in the right hemiscrotum. His parents had first noticed a scrotal mass when he was two years old and was incorrectly diagnosed as hydrocele by an unauthorized practitioner. | 1 |
|  | **3c** | Clinical Findings and Investigations   - In the left hemiscrotum, Doppler confirmed acute epididymitis diagnosis that was treated conservatively with antibiotics and NSAIDs. | 1 |
|  | **3d** | Interventions and Outcome   - In the right hemiscrotum, MRI showed that the lump had separate epididymis and shared a common vas deferens with the right testis, which confirmed the diagnosis of supernumerary testis and the patient underwent a follow-up ultrasound after a month and after six months of his presentation. | 1 |
|  | **3e** | Relevance and Impact   - Polyorchidism is a rare congenital anomaly in the genitourinary tract. It is diagnosed incidentally. Ultrasound or MRI are used to diagnose polyorchidism cases. | 1 |
| **Introduction** | **4** | Background   - Introduction - Polyorchidism or supernumerary testis (SNT) is a rare congenital anomaly in the genitourinary Tract. It is defined as the presence of three testes or more. - Polyorchidism has many forms; Triorchidism (three testes) is the most common type. Bilateral double testis (4 testes) is a rare type(1). - Ahlfeld first described polyorchidism histologically in 1880. It was first reported by Lane in 1895 as clinical case(2). - Rationale - Approximately, there are about 200 cases of polyorchidism in the medical literature(3). - Most cases of Polyorchidism are asymptomatic and are diagnosed incidentally. About 65% of polyorchidism cases were reported on left side with median age 17 years(1). - Ultrasound or MRI help in diagnosis and follow polyorchidism patients(1).   Guidelines and Literature   - 1. Bergholz R, Wenke K. Polyorchidism: a meta-analysis. J Urol. 2009 Nov;182(5):2422–7. - 2. Hassan A, Elhanbly S, El-Mogy MS, Mostafa T. Triorchidism: two case reports. Andrologia. 2014;46(9):1073–7. - 3. Artul S, Habib G. Polyorchidism: two case reports and a review of the literature. J Med Case Rep [Internet]. 2014;8(1):464. Available from: https://doi.org/10.1186/1752-1947-8-464 | 1 |
| **Patient Information** | **5a** | Demographic Details  A 40-year-old man  he works as trader | **2** |
|  | **5b** | Presentation  A 40-year-old man presented to the emergency department complaining of a swelling and pain in the left hemiscrotum with no history of trauma or surgical procedures, therefore he was referred to the urology department. | 2 |
|  | **5c** | Past Medical and Surgical History  with no history of trauma or surgical procedures | 2 |
|  | **5d** | Drug History and Allergies  Drugs and Family history are unremarkable, the patient does not take any medication and he does not recall any type of allergy. | 2 |
|  | **5e** | Family History and Social History  Drugs and Family history are unremarkable, and the patient does not have any concurrent treatments or any Type of allergy. He smokes one pack-year and does not drink alcohol and works as trader. | 2 |
| **Clinical Findings** | **6** | Physical examination indicated an acute left epididymitis. In palpation, we noticed a scrotal mass in the right hemiscrotum | 3 |
| **Timeline** | **7** | His parents had first noticed scrotal mass when he was two years old and was diagnosed incorrectly as hydrocele by an unauthorized practitioner. He had not undergone any further investigation | 3 |
| **Diagnostic Assessment and Interpretation** | **8a** | Diagnostic Assessment  We performed US, which showed normal left testis and enlarged left epididymis with hypoechoic. In addition, we performed Doppler evaluation to demonstrate blood flow, which was increased. Therefore, we confirmed acute epididymitis diagnosis that was treated conservatively with antibiotics and NSAIDs. In the right hemiscrotum, Color Doppler ultrasound evaluation revealed oval lump with  echotexture and blood flow similar to the ipsilateral testis  MRI showed that the lump had separate epididymis and shared a common vas deferens with the right testis, that was not obvious on Doppler ultrasound evaluation, which confirmed the diagnosis of supernumerary testis (Triorchidism). The supernumerary testis had intermediate signal intensity on T1-weighted images and high signal intensity on T2-weighted images and that is the same of normal testes. The left testis measured 43 mm in Long axis, the right testis measured 46 mm, and the supernumerary testis measured 58 mm and situated posteriorly and inferiorly to the right testis.  No Histological examination was performed because the patient refused the biopsy.  The Serum tumor markers were in the normal range. (AFP; 1.44 IU ML−1), and (beta-HCG; 0.5 MUI ML−1). | 3 |
|  | **8b** | Diagnostic Reasoning  No Histological examination was performed because the patient refused the biopsy | 3 |
|  | **8c** | We put Triorchidism and adenomatoid tumor as differential diagnosis for this lump Diagnostic.  The Serum tumor markers were in the normal range. (AFP; 1.44 IU ML−1), and (beta-HCG; 0.5 MUI ML−1).  MRI showed that the lump had separate epididymis and shared a common vas deferens with the right testis. which confirmed the diagnosis of supernumerary testis (Triorchidism). | 3 |
|  | **8d** | Prognostic Characteristics  Then we put the patient on regular follow-up yearly. The patient is still radiologically stable 6 month after his presentation, with his radiologic follow-up the patient has good prognosis, and he did not have any complications as the clinician expected. | 3 |
| **Intervention** | **9a** | Pre-Operative Patient Optimisation  Not Applicable |  |
|  | **9b** | Pre-Operative Patient Optimisation  Not Applicable |  |
|  | **9c** | Specific Details regarding Interventions  Not Applicable. |  |
|  | **9d** | Operator Details and Setting of Intervention  Not Applicable |  |
|  | **9e** | Deviation from Initial Management Plan  Not Applicable |  |
| **Follow-Up and**  **Outcomes** | **10a** | Specify Details regarding the Follow-Up  The patient underwent a follow-up ultrasound after a month and after six months of his presentation. Then we put the patient on regular follow-up yearly. The patient is still radiologically stable 6 month after his presentation, with his radiologic follow-up the patient has good prognosis, and he did not have any complications as the clinician expected. | 4 |
|  | **10b** | Intervention Adherence and Compliance  The patient is still radiologically stable 6 month after his presentation, with his radiologic follow-up the patient has good prognosis, and he did not have any complications as the clinician expected. | 4 |
|  | **10c** | Outcomes  The patient is still radiologically stable 6 month after his presentation, with his radiologic follow-up the patient has good prognosis, and he did not have any complications as the clinician expected. | 4 |
|  | **10d** | Complications and Adverse Events  he did not have any complications as the clinician expected. | 4 |
| **Discussion** | **11a** | Strengths  Triorchidism is the most common type of polyorchidism. However, bilateral double polyorchidism (4 testicles) had been reported(6). The left side is the most common place for supernumerary testicle, inguinal canal is also a possible location(1). In comparison, the supernumerary testicle in our patient is in the right hemiscrotum.  The median age of polyorchidism cases is 17(1) and approximately, there are about 200 cases of polyorchidism in the medical literature(3).  Here we present a case of 40-year-old man who was diagnosed incidentally with polyorchidism in the right hemiscrotum which is quite unusual in this age.  In our case, the classification of the Supernumerary testis was type A2 according to Bergholz and Wenke (2009) classification and type C according to Leung classification.  In the same meta-analysis, 73% of the cases were diagnosed histologically which is quite different in our case where we only diagnosed the case with imaging studies(1). this also carry a less aggressive approach to the patient. | 4 |
|  | **11b** | Weaknesses and Limitations  We did not confirm the diagnosis by histological examination because the patient refused the biopsy and there is no need for it because there was no neoplastic signs and the imagining study alone confirmed polyorchidism(1). | 5 |
|  | **11c** | Relevant Literature   - Based on Leung classification there are four types of polyorchidism: Type A. the supernumerary testicle has neither epididymis nor vas deferens and has no connection to the other testicles. Type B. the supernumerary testicle shares the same epididymis and vas deferens of the other testicles. Type C. the supernumerary testicle has separate epididymis but shares a vas deferens with the other testicles. Type D. the supernumerary testicle has separate epididymis and vas deferens. (7) - Furthermore, in a meta-analysis study another classification has been described: Type A when it is connected to vas deferens with subgroups: A1 separate epididymis and vas deferens. A2 separate epididymis but shares vas deferens with other testicle A3 shares epididymis and vas deferens with other testicle. A4 separate vas deferens but shares epididymis with other testicle. It is classified as Type B when it has no connection to vas deferens with subgroups: B1 separate epididymis. B2 only testicular tissue. The most common type is A3(1). | 5 |
|  | **11d** | - , when the patient is asymptomatic Type A (our patient) the concentrative treatment is recommended, but the patient should be monitored regularly because there is an increased number of malignancy in case of polyorchidism. | 6 |
|  | **11e** | Take-Away Lessons  The treatment of polyorchidism is still debated. In the past, Surgical removal of supernumerary testicle was preformed but the presence of imaging study changed the way we treat polyorchidism, when the patient is asymptomatic Type A (our patient) the concentrative treatment is recommended, but the patient should be monitored regularly because there is an increased number of malignancy in case of polyorchidism. If the malignancy was confirmed a surgical removal is recommended(1). Based on that our patient is under concentrative treatment with close regular monitoring. | 6 |
| **Patient Perspective** | **12** | - Where appropriate, the patient should be given the opportunity to share their perspective on the intervention(s) they received (e.g. sharing quotes from a consented and anonymised interview). |  |
| **Informed Consent** | **13** | Written informed consent was obtained from the patient for publication of this case report and accompanying images. A copy of the written consent is available for review by the Editor-in-Chief of this journal on request. | 6 |
| **Additional Information** | **14** | **Competing interests**  Authors declare they have no conflict of interest.  **Funding**  No funding was obtained for this case report.  **Acknowledgments**  We would like to acknowledge Bader Ammory for proofreading the manuscript and Muhammad Nour Sa'adi for technical support.  **Authors' contributions**  AK and MEJ: Revision of the manuscript. MM, MZBA and BB: Drafting of the manuscript. OA: Conception and design of the study. AK: Approval of the final version of the manuscript.  All authors confirm that they have read and approved the final manuscript. | 7 |
| **Clinical Images and Videos** | **15** | - Where relevant and available, include clinical images to help demonstrate the case pre-, peri-, and post-intervention (e.g. radiological, histopathological, patient photographs, intraoperative images). - Where relevant and available, include a link (e.g. Google Drive, YouTube) to the narrated operative video can be included to highlight specific techniques or operative findings. - Ensure all media files are appropriately captioned and indicate points of interest to allow for easy interpretation. |  |
| **Referencing the Checklist** | **16** | - This case report has been reported in line with the SCARE Criteria(4). | 2 |
